# The Book World of Medicine and Science

**Published:** 1902-01-11

**Authors:** 


### The Book World of Medicine and Science.

Syphilis and other Venereal Diseases. By H. De Méric, M.R.C.S. (London: Baillière, Tindall and Cox. 1901. Price 5s. net.)

Mr. De Méric has succeeded in condensing into a small volume a very large amount of useful information in regard to the group of diseases which go by the name "venereal." He begins by pointing out that although syphilis and the so-called "soft" sore are absolutely distinct diseases considerable difficulty is often met with in their diagnosis in their early stages, and he shows how misleading and unscientific is the division into "soft" and "hard" sores. He would do away with these terms and replace them by the more definite expressions "simple" and "syphilitic." In making a diagnosis between simple and syphilitic sores great importance is attached to the condition of the inguinal glands, as to which distinctions are laid down with a definiteness which is more sharply drawn than may perhaps be acceptable to some. He says that we find "in simple sores either nothing at all in the groin or intense inflammation of the lymphatic glands, speedily going on to suppuration and abscesss. In syphilitic chancre the glands in one or both groins are always indolently enlarged and hardened, acute inflammation and suppuration in them being quite the exception." Further, it is stated that although in syphilitic cases suppuration may appear imminent and yet be avoided by rest and proper local applications "this would never happen in a case of simple sores, in which any inflammation in the inguinal glands is certain to run on to suppuration and abscess." According to this teaching a bubo, arising from a venereal sore, which fails to suppurate and produce abscess is of necessity syphilitic—a doctrine which will certainly not receive universal acceptance.

In regard to the much-discussed question of the origin of the so-called *bubon d'emblée* Mr. De Méric gives no adhesion to the theory that venereal poisons can be absorbed during connection, and, passing through the lymphatics of the penis without producing a sore, proceed to determine a swelling in the groin, which may or may not suppurate. He points out that any exercise unduly prolonged may cause the glands to enlarge, and that in tuberculous persons the lymphatic glands are peculiarly susceptible to the effects of irritation. Indeed he seems inclined to regard tuberculosis as being at the bottom of many of the cases which some surgeons describe as *bubons d'emblée*.

In speaking of the diagnosis of syphilis we are cautioned not to attribute too much importance to the hardness of the sore, for in some, especially in the "small herpetical-like sore," it is often very difficult to detect any induration. We are also told that "the fact of the syphilitic chancre being invariably *single* has been too much insisted on." We often see several primary lesions. The great point is that these sores do not and never can increase in number, a syphilitic sore can never by infecting a fresh spot produce a fresh sore on the same patient, as constantly happens with patients suffering from a simple sore. The great diagnostic point, however, lies in the glands of the groin. The surgeon "may be puzzled about length of incubation, number of sores, induration, etc., but in the groin he will find the key to the difficulty of giving a definite opinion as to whether the chancres are simply local or syphilitic. With the syphilitic chancre there is *always something* in the groin: the glands are *always* enlarged, the enlargement being generally indolent." There is a difficulty, however, in regard to certain cases in which there may be enlarged indolent tuberculous glands in combination with a few spots of herpes on the genitals.

The chapters on treatment are thoroughly practical, and will be read with much interest as being the outcome of a wide experience. Much is very usefully said about the desirability of rendering the treatment as little unpleasant as possible, a point of considerable importance, for, as Mr. De Méric remarks, the surgeon will rarely be able to persuade the patient to continue the treatment for months and months after all visible symptoms have disappeared unless that treatment be, comparatively speaking, easy and pleasant. The book is hardly a scientific treatise, but it is an eminently practical handbook.

Elementary Practical Hygiene (Section I.). By William S. Furneaux. (London: Longmans, Green, and Co. 1901. Pp. 233, with 146 illustrations. Price 2s. 6d.)

Hygiene must be regarded not so much as a distinct special subject as a collection of scientific facts which have special reference to health, and the object of the work before us "is to provide the student with a practical guide to elementary physics and chemistry as far as is necessary to form a sound basis for the study of domestic science." By means of simple experiments the student is recommended to verify the text for himself, and at the end of each chapter is a useful summary of its contents. After a description of the measurements of length, area, volume, and mass, the reader is introduced to the effects of heat and instructed in the use of thermometers and the scientific meaning of temperature. Following this is an account of the atmosphere and the effects of combustion, leading up to a practical discussion on ventilation and warming. Several experiments with carbonic acid gas are followed by a description of the chemistry of the human body and the fate of the food stuffs. Here the author apparently makes a distinction between muscle and meat, which is apt to be misleading, for he remarks that we derive our nitrogenous foods from the "*albumen* of eggs and meat, *myosin* of muscle, *fibrin* of meat, and *casein* obtained from milk." Lean meat is of course all muscle and nothing else. We are next given an account of the digestive organs of mankind illustrated by diagrams of the teeth, stomach, intestines, and the glands which secrete the digestive juices, but it is doubtful whether the drawings of a highly magnified section of peptic glands from the stomach and of a villus of the intestine will be readily appreciated by the readers of a work of this kind. Many useful practical hints are given regarding cooking, the preservation of foods, washing, and the removal of stains by soda and soap, and we are also instructed as to what takes place in the process of fermentation of wine and in the action of yeast. The work concludes with a chapter on disinfectants, deodorants, and antiseptics. It will thus be seen to deal with a vast range of subjects, the whole of which are carefully and accurately treated, so that as an elementary textbook it can be safely recommended to any young student.

